# Movement of Free-Ranging Koalas in Response to Male Vocalisation Playbacks

**DOI:** 10.3390/ani12030287

**Published:** 2022-01-24

**Authors:** Alex Zijian Jiang, Peter Murray, Clive Phillips, Andrew Tribe, William Ellis

**Affiliations:** 1 School of Veterinary Science, The University of Queensland, Gatton 4343, Australia; zijian.jiang@uqconnect.edu.au (A.Z.J.); peter.murray2@usq.edu.au (P.M.); 2 School of Agriculture and Environmental Sciences, University of Southern Queensland, Toowoomba 4350, Australia; 3 Sustainability Policy Institute, Curtin University, Bentley 6102, Australia; clive.phillips@curtin.edu.au; 4 Institute of Veterinary Medicine and Animal Sciences, Estonian University of Life Sciences, Kreutzwaldi 1, 51014 Tartu, Estonia; 5 Turner Family Foundation, Hidden Vale Wildlife Centre, 617 Grandchester Mount Mort Rd., Grandchester 4340, Australia; andrew.tribe@turnerfamilyfoundation.com.au; 6 School of Agriculture and Food Sciences, The University of Queensland, St Lucia 4072, Australia

**Keywords:** *Phascolarctos cinereus*, marsupial, social structure, communication, mating strategy, behavioural ecology, reproductive behaviour, wildlife conservation

## Abstract

**Simple Summary:**

Conservation management is critical for threatened wildlife species and an effective conservation approach relies on good understanding of animal behaviour in natural habitat. Currently, koalas are listed as vulnerable to extinction in Australia with declining populations, but their social and breeding system remains unclear. Male koala vocalisations, known as bellows, are believed to be closely related to their breeding behaviour. Bellows incorporate callers’ body size information which can be perceived by other koalas. In this study, we tested the behavioural responses of 20 GPS-collared free ranging koalas to bellow recordings collected from small (<6 kg) and large (>8.5 kg) adult male koalas. We report evidence of intra-male competition, with adult males approaching bellow playbacks, particularly those from small-sized males. In contrast, juvenile males under three years of age showed avoidance of the playbacks. No patterns in the response of females were detected. Our results provide the strongest evidence yet that bellows are primarily a means by which males occupy and control habitat space during the breeding season. Future studies are required to see if female response to bellows depends on their reproductive status.

**Abstract:**

Effective conservation strategies rely on knowledge of seasonal and social drivers of animal behaviour. Koalas are generally solitary and their social arrangement appears to rely on vocal and chemical signalling. Male koala vocalisations, known as bellows, are believed to be closely related to their breeding behaviour. Previous research suggests that oestrous female koalas use bellows to locate unique males to mate with, and that males can similarly use bellows to evaluate the physical attributes of their peers. We tested the behavioural responses of 20 free ranging koalas to bellow recordings collected from small (<6 kg) and large (>8.5 kg) adult male koalas. Individual koala movement was reported by hourly-uploaded GPS coordinates. We report evidence of intra-male competition, with adult males approaching bellow playbacks, particularly those from small-sized males. In contrast, males under three years of age were averse to the playbacks. No patterns in the response of females were detected. Our results provide the strongest evidence yet that bellows are primarily a means by which males occupy and control space during the breeding season. Future studies are required to see if female response to bellows depends on their reproductive status.

## 1. Introduction

Vocalisations have been found to serve a number of purposes; for example, they facilitate recognition of kin [1,2,3,4,5,6,7], warn of predators [8,9], confirm territories [10,11], solicit sexual attention from conspecifics [12,13,14] and permit assessment of rivals [15]. Koalas, *Phascolarctos cinereus*, are vocal marsupials, using bellows to communicate in vast Australian eucalypt forests. They use a novel vocal organ, an extra pair of vocal folds [16,17], to produce extra low-pitched vocalisations. Bellows are unique and reliable signals of size in koalas [17]. Bellows are typically produced by male koalas during their breeding season (southern hemisphere spring through summer) [18,19,20], peaking in frequency during mating activity [21]. Koala bellowing has been associated with movement [20] and intraspecific interactions [19].

The definitive function of bellows in koala behaviours is, however, unclear and believed to influence spatial and social arrangement [20,22,23,24]. Captive oestrous female koalas appear more interested in the bellows of larger males, spending longer periods of time facing and/or approaching speakers that broadcast bellows of lower formant frequency [25], which suggests a role for bellows in sexual selection. However, field based paternity analysis has revealed that male body size and reproductive success are not linearly correlated over time [26] and the role of resident males as arbiters of sexual selection in koala society [20,27] is not confirmed in genetic studies [28,29]. Nevertheless, the observations of the bellow preference in captive oestrous females [25,30] provide the impetus for testing the hypothesis in free-ranging koalas that bellows associated with different male koala body sizes will elicit specific and detectible behavioural responses from females, and also possibly from males.

Koalas are solitary animals but with often overlapped home range [20]. Female koalas move further and more often when bellows are more frequent in the environment [29] and interactions between females also increase during the breeding season [21]. Male-male physical interactions are less frequent but of longer duration than female-female interactions in lower density populations [21], but more frequent in high density populations [31] leading the later study to suggest structural plasticity [32] with population density as the influential factor across koala populations [31].

Despite the evidence that bellows are a reliable indicator of body size in male koalas [17] and findings of female preference for the bellows of the large males [25], the relationship between body size, sexual selection and reproductive output is unclear. Sires have been found to be larger than non-sires [26], but no relationship between male koala body size and the frequency of male-female interactions in either low [21] or high koala-density areas [31] has been detected, and body size was reported to account for only 13% of the variation in male reproductive output in the study of Ellis and Bercovitch [26].

Although informed by research on koalas in captivity, significant gaps remain in our knowledge of the role of bellows in free-ranging populations. Social dynamics of animals in captivity can differ from those in wild conspecifics [33], so field-based investigations are required. Our aim was to investigate the impact of bellows on the spatial arrangement of koalas by broadcasting bellows recorded from large and small koalas in a field setting. Our testable hypothesis was that female koalas would be attracted to bellows of larger males, whereas males would avoid the bellow of a larger individual. We sought to determine whether free-ranging koalas actively search for males by attending to their bellows, and whether we could distinguish any preference amongst koalas for body size of the recorded bellower.

## 2. Materials & Methods

### 2.1. Study Site

**Figure 1:**
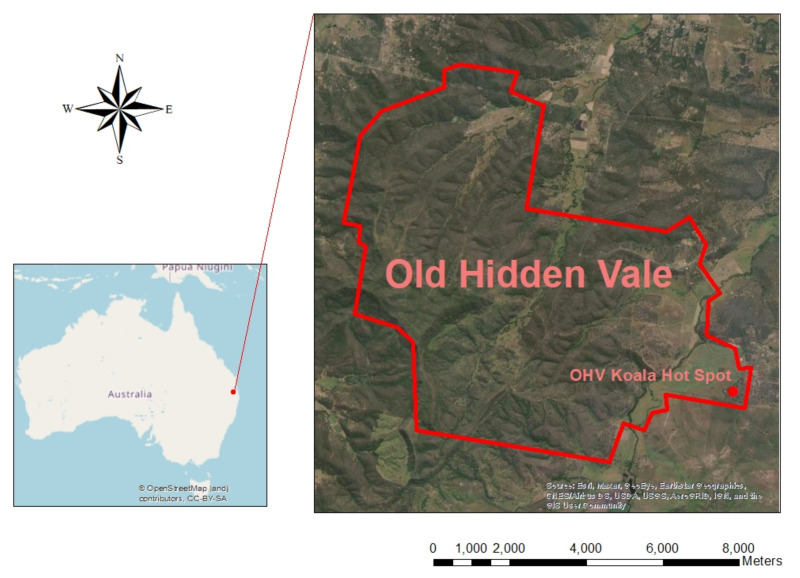
Map of Old Hidden Vale with the location of the Koala Hot Spot.

This study consisted of two koala bellow playback experiments: an individual bellow test and a group bellow test (described below). Tests were conducted on private bushland located at Old Hidden Vale (OHV; 27°43′ S, 152°27′ E), in south-east Queensland (Figure 1). The Old Hidden Vale property is 4858 ha, with about 70% covered by remnant open forest dominated by Regional Ecosystem (RE) 12.9–10.2, 12.9–10.7, 12.8.16 and 12.3.3 [34] which is considered high-quality koala habitat with koala fodder trees such as *Eucalyptus tereticornis* Sm., *E. crebra* F. Muell and *Corymbia citriodora* (Hook.) K.D. Hill and L.A.S. Johnson [35]. The individual test was conducted using koalas that were distributed across the eastern half of OHV. The group test was conducted in an alluvial allotment on the eastern side of the property (OHV Koala Hot Spot) which, based on a systematic scat survey in 2018 [36], has the highest koala density on OHV (0.32 koalas per ha).

### 2.2. Study Animals and Data Collection

Eight male and 12 female adult koalas (all over one year old) were captured using standard “flagging” techniques [37] and collared (VHF/GPS—LX telemetry monitoring system (LX GROUP™, Sydney, NSW, Australia)) in 2018. Twelve (four males and eight females) koalas were resident across the eastern half of OHV property, and eight (four males and four females) were residents of a higher-density group at the OHV Koala Hot Spot. Individual bellow tests were conducted by broadcasting recordings, on an individual basis, to each of the 20 koalas, and group bellow tests were separately conducted by common broadcasting to the group of eight koalas in the OHV Koala Hot Spot. Generally, koalas become independent at one year old, then females are sexually mature, but males are believed to start to breed a few years after independence [18,38]. Therefore, in this study, koalas were distinguished into classes of adult male (MA, *n* = 7)—males over three years old, juvenile male (MJ, *n* = 2)—males under three years old, single female (FS, *n* = 9)—females without any dependent joeys, and female with dependent joey (FDJ, *n* = 3). One male koala grew to over three years old during the tests, so we included his pre-three years old data in juvenile and post-three years old data in adult. In this study, oestrous females were not identified. Currently, determination of oestrous in female koalas relies on observing oestrous behaviour (in captivity), such as bellowing and mounting, combined with the morphology of external genitalia and blood hormone tests [20,25,39,40,41,42]. Oestrous behaviour stops directly after mating, and lasts 10 days if there is no copulation [39]. Therefore, it is extremely difficult to predict or determine oestrous in female koalas in a free-ranging environment. Hence our classes, that were based on the presence or absence of young, allowed an unequivocal distinction for this study.

Koala collars were programmed to upload GPS location hourly (during the bellow playback tests) or 12-hourly (outside of test periods, at 10:00 and 22:00 daily). The LX collars also reported koala activity levels, using an inbuilt accelerometer to detect and report movement and inactivity in hourly (or 12-hourly, non-test periods) intervals [43]. Koalas were recaptured every 1–3 months to monitor health, reproductive status and collar fit.

### 2.3. Bellow Recordings and Broadcasting

Koala bellows were recorded from six captive male koalas: four from Australia Zoo (Beerburrum) and two from RSPCA Queensland Wildlife Hospital (Wacol) in southeast Queensland. Bellows chosen for tests were of 30 s duration, which is the average bellow length of koalas in Queensland [19,29]. They were recorded from three males of less than 6 kg (“small male bellows”), and three males of over 8.5 kg (“large male bellows”). The six bellow recordings were paired (one small and one large bellow) to present three replicates for broadcast purposes (Table 1). In the field, bellow recordings were broadcast using speakers (Chiayo™ Focus505, Taipei, Taiwan, China) set at one metre height above the ground. The volume for all tests was set to 76 decibels, on average, measured by an Apple™ iPhone 6s (Cupertino, California, CA, USA) with the Decibel X™ (SkyPaw Co., Ltd., Hanoi, Vietnam) application, at a distance of one metre, reported as consistent with naturally produced koala bellows [44].

### 2.4. Individual Responses to Bellows

Between October 2019 and November 2020, 49 individual tests were conducted on 20 koalas (7 MA, 2 MJ, 9 FS, 3 FDJ) on OHV. Each koala was tested between one and four times with various bellow sets and playback sequences. When any two koalas were within 300 m of each other, as well as when multiple tests were conducted on one koala, the time interval between tests was at least one week to minimise the probability of artefacts such as habituation influencing their responses.

During each test, a large and a small bellow were broadcast, from opposite directions, at a distance of 50 m from the subject koala. One speaker played three small-male bellows and the other three large-male bellows. Small and large bellows were played alternatively with a five-minute interval between each bellow. This interval was consistent with koalas responding to each other’s calls in the wild [24]. The initial bellow, small or large, was alternated between tests to eliminate any sequence effect. The three bellow sets were also alternated to minimise bellow source bias.

**Figure 2:**
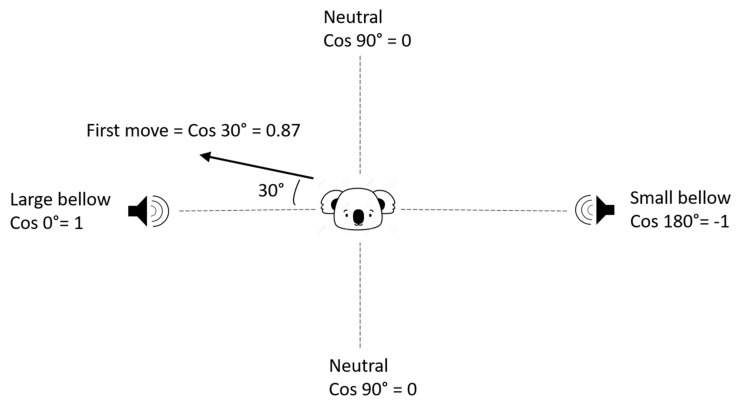
An example of how the value for ‘first move direction’ was calculated.

Observations of koala behaviour (“first look” and/or “first move”) during and after the playback experiments were made. The “first look” was the duration of a koala’s first head turn towards the speaker broadcasting the bellow [44], which started when a koala turned its head towards the speaker (if previously facing away) or the onset of playbacks if a koala was already facing towards the speaker when the playback started, and ended when the koala turned its head away from the source for more than 10 s. The first look response was observed from at least 30 m away using Bushnell™ (Overland Park, Kansas, KS, USA) 10 × 42 binoculars and also recorded on a Canon™ (Tokyo, Japan) EOS 5D Mark II camera. The “first move” was the first detectable change of trees by a koala (as reported by the LX monitoring system) before 10:00 on the day following the bellow test. Koalas that did not move after the test, before 10:00 on the next day (Stay On Site: “SOS”), were those that either remained in the same tree, or were reported to move a distance shorter than could be verified according to our GPS accuracy (described below). The first recorded move included both compass direction and distance. The direction of move was calculated as the angle of movement (degrees from 0°—which was the direction of the “large” bellow speaker) of any koala move. The large bellow direction was 0°, the small bellow direction 180° and a neutral direction 90° which was towards neither the small nor large bellow (i.e., in a perpendicular direction to the Small-Large axis). In order to meet the normality assumption required by the linear mixed-effects model, we then took the cosine value of the angle, converting the direction of the first move into a scale from −1 to +1, in which −1 (cosine 180°) represented the small bellow direction, +1 (cosine 0°) represented a large bellow direction, and 0 (cosine 90°) represented a neutral direction. The closer the value was to +1 or −1, the closer the movement direction was towards the large or small bellow broadcast speaker. For example, if a koala moved towards a direction of a 30° angle from the large bellow speaker, it was quantified as 0.87 (Figure 2).

To control for natural directional tendency in koala movement, we examined the koala move direction and distance before each bellow test. These control data were obtained from subject koalas on the day prior to the bellow tests, so that other variables such as time of day, temperature and season, that may have impacted koala behaviour, were accounted for. These data were collated as a “control group”, representing the baseline or pre-test behaviour of the koalas in the experiment to detect any underlying directionality of movement. The direction of koala moves in the control group was calculated as for koalas in the bellow tests: the control group however was subject to a virtual bellow test with speakers playing no sound, placed in the same directions of those in the actual bellow test.

### 2.5. Group Responses to Bellows

**Figure 3:**
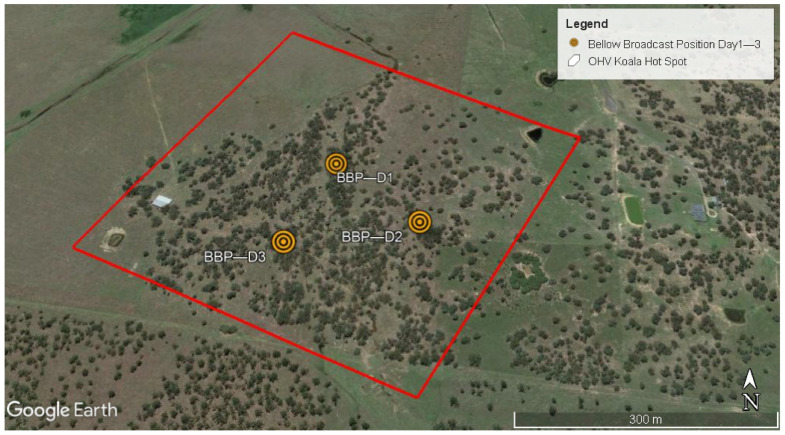
OHV Koala Hot Spot and Bellow Broadcast Positions (BBP) for testing broadcast koala bellows to groups of koalas on Day 1 to Day 3. Map image generated from Google Earth (https://earth.google.com, accessed on 1 January 2022).

**Figure 4:**
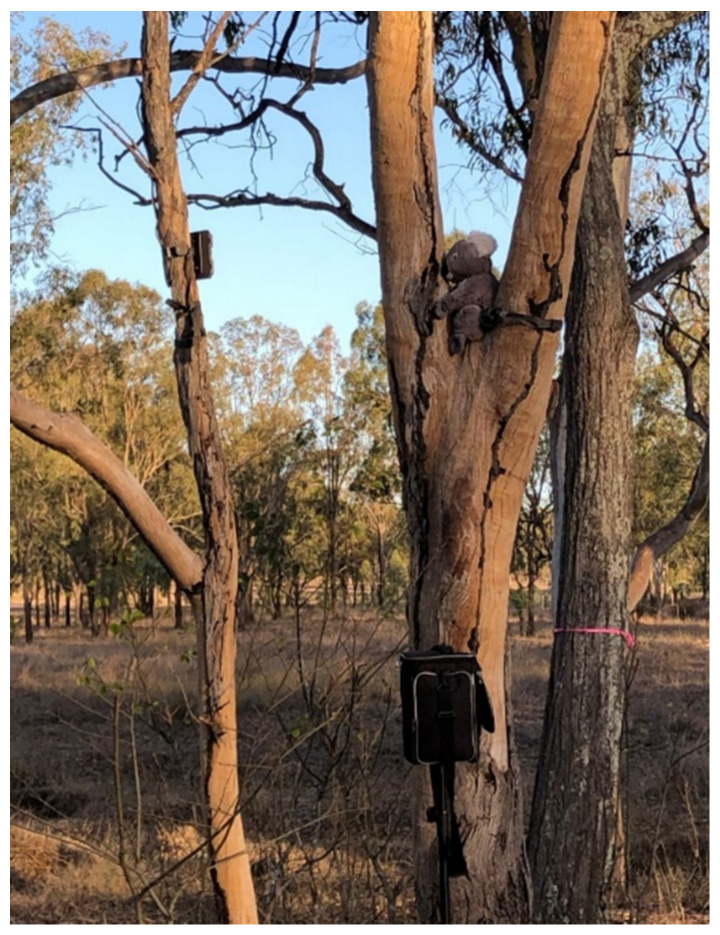
Group bellow test settings with the koala model, camera and speaker used to broadcast the bellows in the field.

Individual bellow tests were used to investigate individual koala preference for large or small bellows, but we also conducted a “group” bellow test to investigate whether koala movement was more generally influenced when only one type of bellow was broadcast. This test was conducted using eight koalas at the OHV Koala Hot Spot, from November 2019 to April 2020. Three bellow broadcast positions were pre-selected to evenly cover the study area (500 × 500 m), approximately 200 m apart (Figure 3), in accordance with the reported active space of a male bellows (100–150 m) [45]. Only one type of bellow, either small or large, was played at a time. A model koala sprayed with koala faeces was placed in a tree fork adjacent to the speaker for the group tests, to provide a standardised visual (and potentially olfactory) environment (Figure 4). Infrared video cameras (Swift Enduro™ trail camera, Toowoomba, Queensland, Australia) were trained on the koala model to record any interactions with free-ranging koalas.

The group bellow test incorporated three separate protocols (Table 2): a model test (koala model but no speaker), a one-hour evening bellow test (large or small bellows with the koala model and speaker) and a six-hour overnight bellow test (large or small bellows with the koala model and speaker). There was also a control round before each test, which was a virtual test round without any koala model or speakers in the week before those three tests. Each test round lasted three days, during which the speaker and koala model, if applicable, were set at one broadcast position for one day, then switched to the next position, until all three bellow positions had been tested. In the model test round, only the koala model was set (without a speaker) at each bellow position, at 18:00 each day, to control for the possible visual and olfactory effects of the model itself. In the evening and overnight bellow test rounds, small or large koala bellows were broadcast at each bellow playback position every 15 min starting from 18:00, for one and six hours each night, respectively. There was at least a one-week interval between each round to avoid habituation artefacts.

### 2.6. GPS Accuracy and the First Move Determination

**Figure 5:**
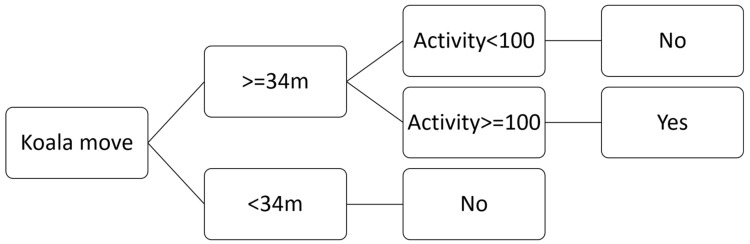
Koala first move decision tree based on the GPS fix distance and activity level reported by the LX system.

Due to inherent errors, consecutive locations reported by stationary GPS units can be widely separated. For our study, we excluded from koala movement data any reported movement distance below a threshold that corresponded to our estimated uncertainty, determined from our stationary tests. The stationary tests consisted of deploying two fixed-position LX collars; one on top of a hill and the other on an alluvial plain on the property. The deployments were for two weeks and generated 220 GPS fixes. The distance between pairs of consecutive fixes (indicative of “false” movement) ranged from 0 to 80 m (*X* + SE = 12 + 0.70 m, *n* = 218, median = 10 m), with 95% of values below 34 m. The LX system does not report estimated accuracy, so no correlation between estimated and actual accuracy was possible. However, our results indicate that applying a threshold of 34 m would assign movement to a stationary koala with a probability of only 5%. Although it is also likely that we excluded some small movements by koalas (<34 m), this approach provided confidence that reported movements in our dataset were not GPS location error. In addition, we also referred to the accelerometer data which recorded activity of the collared koalas. Observed koala movement between trees was, without exception, associated with an hourly accelerometer activity level greater than 100. Hence, during our bellow tests, we excluded from our data set any reported movement with either a distance below 34 m or an activity level below 100 (Figure 5).

### 2.7. Statistical Analyses

All factors, and their interactions, that could have impacts on koala’s responses to bellow playbacks, including season, playback sequence and source, and koala’s sex, age, body mass and class, were tested as covariates in the models used for each test.

#### 2.7.1. Individual Responses to Bellows

In order to test whether koalas spent different periods of time looking at, and approaching, small or large bellows, the duration of their first look (log_10_ transformed to meet the normality assumption of linear mixed model, Shapiro-Wilk normality test *p* = 0.53), the direction (from small bellow −1 to large bellow +1) and distance (log_10_ transformed to meet the normality assumption, Shapiro-Wilk normality test *p* = 0.21) of their first move after individual bellow tests, were analysed via linear mixed models [46] with individual koalas as a random effect, α = 0.05, using lme4 [47] and lmerTest [48] packages in R version 3.5.1 (R Foundation, Vienna, Austria) [49]. Factors affecting whether koalas either stayed on site or moved were assessed using a generalised linear mixed model [46] with a binomial distribution. We also treated the effect of individual koalas as random, using data from the previous day as their control.

#### 2.7.2. Group Responses to Bellows

To analyse the time that koalas spent near the speakers broadcasting bellows, we counted the number of hourly GPS fixes of koalas that were located inside a 100-m radius of the broadcasting speaker within 24 h of the bellow commencement (each day at 18:00), together with the total fixes, in each test round. We used a linear mixed model with individual koalas as a random effect, similar to the model used in the individual bellow test, to test the proportion of fixes located in the 100-m radius range, as well as the hourly activity level of koalas during the same period. In addition, approach behaviour (when koalas moved from outside into inside the 100-m radius circle around the speaker) and avoidance behaviour (when koalas that were inside the 100-m radius circle before test moved out of the circle after the test commenced) were also recorded and analysed with a generalised linear mixed model with binomial distribution.

## 3. Results

Koala response behaviour towards bellow playbacks was primarily impacted by koala’s class (i.e., adult male, juvenile male, single female, and female with a dependent joey), whereas other factors including the animal’s sex, age and body mass, breeding season, playback source and sequence had no significant effects on their behaviour.

### 3.1. Individual Bellow Tests

#### 3.1.1. Move Direction

**Figure 6:**
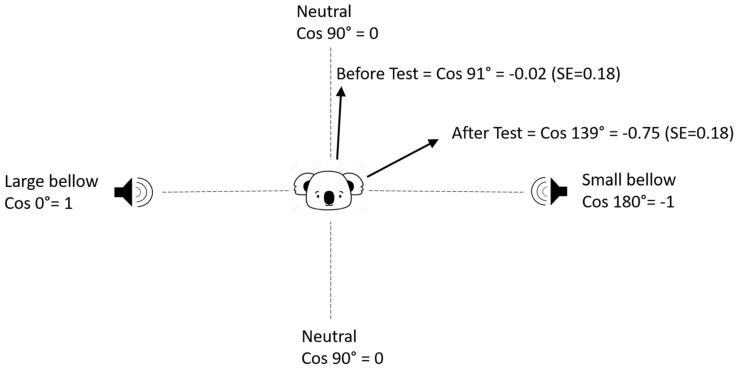
The change of the mean first move of adult male koalas before and after bellow tests.

**Figure 7:**
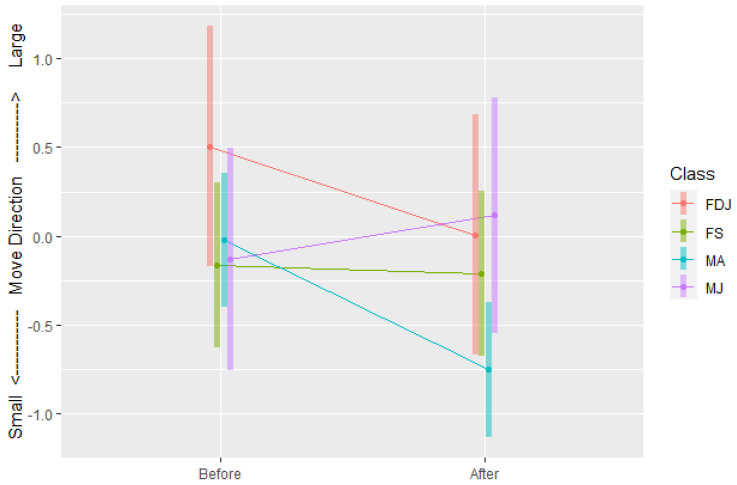
The direction (cosine transformed degree) that adult male (MA), juvenile male (MJ), single female (FS) and female koalas with a dependent joey (FDJ) moved before and after the bellow tests.

The direction of the koala’s first move in the individual bellow test was influenced by bellow playbacks combined with the class of koalas (interaction effect *F*_3,47_ = 3.06, *p* = 0.03). The first move direction of adult males (males over three years old) was significantly altered from the near neutral direction (91° from large bellow) in the control before the bellow test, and towards the small koala bellow direction (139° from large bellow) after the bellow test (Figure 6 and Figure 7). This was not observed for juvenile males nor females (single and with a joey). There were in total 19 tests on seven adult male koalas with valid hourly GPS data, in which 68% (*n* = 13) detected movement towards the small bellow (135–180°), 16% (*n* = 3) were neutral (45–135°), 10% (*n* = 2) stayed on site (no movement) and only one koala moved in the direction of the large bellow (0–45°). Anecdotally, of the seven adult males, which were all tested two to four times, four invariably approached the small bellows (11 tests total) but the response was inconsistent for the other three koalas. Two adult male koalas were captured on film descending their trees during the bellow broadcast and moving towards, then staying near, the small bellow speaker. Juvenile (under three years old) male koalas did not approach any bellows. One male started to approach the small bellows after he was three years old.

#### 3.1.2. Move Distance

**Figure 8:**
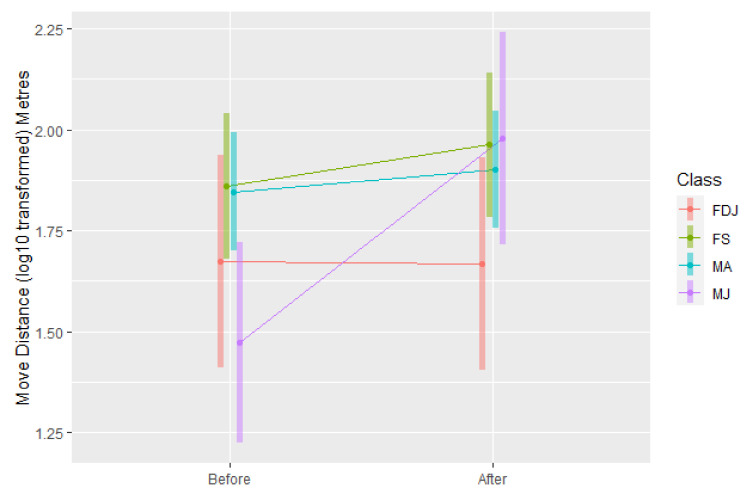
The distance (log_10_ transformed metres) that adult male (MA), juvenile male (MJ), single female (FS) and female koalas with a dependent joey (FDJ) moved before and after bellow tests.

There was a significant interaction effect of bellow test combined with the class of koalas (*F*_3,46_ = 2.97, *p* = 0.04) on the first move distance. In particular, juvenile males, i.e., young male koalas less than three years old, travelled significantly further in their first move after the test (Figure 8).

#### 3.1.3. Stay on Site

Of the 49 individual bellow tests, koalas were recorded as staying on site after the bellow test on eight occasions (five females and three males). There were no significant effects of bellow playbacks (*X*^2^_1_ = 3.33, *p* = 0.08) or class (*X*^2^_3_ = 4.04, *p* = 0.26) on whether koalas stayed on site or moved.

#### 3.1.4. Look Response

**Figure 9:**
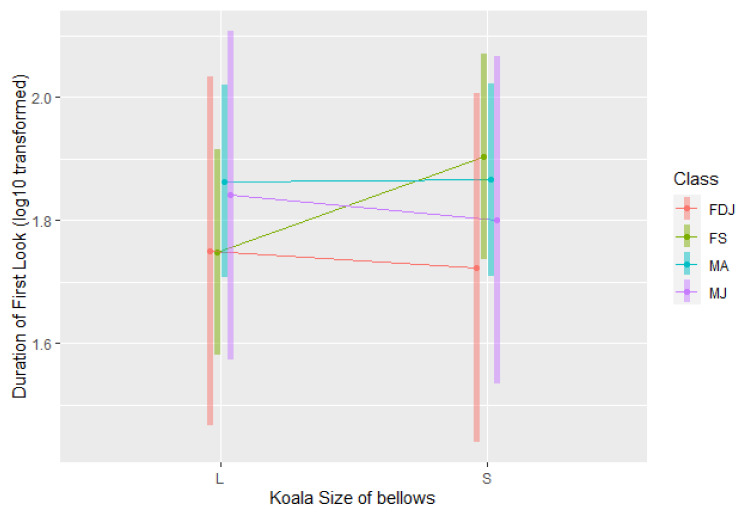
The difference in first-look response of adult male (MA), juvenile male (MJ), single female (FS) and female koalas with a dependent joey (FDJ) towards small (S) and large (L) koala bellows.

There were no significant differences between koalas’ first look response towards large and small male bellow playbacks (*F*_1,44_ = 0.48, *p* = 0.49). No interaction effects of bellows combined with the class were detected (Figure 9).

### 3.2. Group Bellow Tests

#### 3.2.1. Activity Levels

The bellow playbacks had a significant impact on koala activity levels (*F*_2,111_ = 3.95, *p* = 0.02), with an increased activity level during bellow broadcasting rounds compared with control rounds (without bellows) (Table 3). In the koala model round when no bellows were involved, the koala activity level was not affected by the presence of the model without bellows (*F*_1,24_ = 2.03, *p* = 0.17).

#### 3.2.2. Time Stayed near Speakers Broadcasting Bellows

The occurrence of bellow playbacks and koala class had interaction effects on the time that koalas stayed near (within 100 m) the source of the bellows (*F*_6, 132_ = 2.42, *p* = 0.03). In particular, the duration of stay for adult males near the speakers broadcasting small bellows was significantly longer than for the same animals near the speakers broadcasting large bellows, while both were longer than control rounds without bellows (Table 3). As direct field evidence, vision of adult male koalas climbing into the tree where the “bellowing” koala model sat was recorded. Time spent by the other koalas staying near speakers appeared unaffected by the bellows. In the koala model round when no bellows were broadcast, the time spent by koalas staying near the model was not affected by the presence of the koala model without bellows (*X*^2^_1_ = 0.006, *p* = 0.94).

#### 3.2.3. Approach and Avoidance Behaviour

The frequency of approach behaviour by koalas was impacted by bellow playbacks (*X*^2^_2_ = 8.89, *p* = 0.01) as well as the class of koalas (*X*^2^_3_ = 10.8, *p* = 0.01), with more approaches occurring with adult male koalas and during bellow broadcasting compared with the control (no bellows). Koala avoidance behaviour was not affected by bellow playbacks (*X*^2^_2_ = 2.17, *p* = 0.34) (Table 3).

## 4. Discussion

The aim of this study was to investigate how the behaviour of free-ranging koalas was affected by broadcasts of bellows from small and large male koalas. The results demonstrated that dependent on the koala’s class, bellow playbacks significantly affected koala movement (direction and distance), activity level, and time spent near speakers.

### 4.1. Adult Males

In the individual bellow test, compared with the control of bellows not broadcast, moving directions of adult male koalas (males older than three years old, *n* = 7), were significantly affected by bellow playbacks and altered towards the direction of small male bellows. Adult males moved directly towards the small bellow in 68% of tests (*n* = 19). In the control environment (before the bellow tests), the average moving direction of koalas approached neutral, indicating movement toward neither playback location but instead perpendicular to the small-large bellow speaker line. This indicates that koala move directions before the bellow broadcast tests, were not naturally directed toward either speaker location. After koala bellows were broadcast, adult male koalas turned their average move direction to −0.75, a direction of 41° (180–139°) from the small koala bellow speakers (as shown in Figure 6), while other koala movements remained neutral. This indicates that after hearing the bellows of small and large koalas from two opposite directions, adult male koalas were inclined to approach the small male bellows, while other koalas appeared unaffected in their move direction. The adult male response was not only supported by the statistical analyses above, but also demonstrated by field observations that, immediately after hearing both bellow playbacks, two adult males descended their tree and approached the source of the small bellows.

The group bellow test was designed to detect subtle but universal responses within groups of koalas and to establish whether a bellow broadcast would affect multiple koalas within any given population. The results confirm a natural drive or overall response (perhaps detecting fear or attraction) governing the behavioural patterns of adult male koalas we detected in the individual bellow tests. When exposed to the bellows of both small and large males, adult male koalas approached the small male bellows in individual tests. We hypothesized, based on our individual tests that males were either a) attracted to the bellows of small males or b) avoiding the bellows of large males. This dilemma was resolved during the group bellow test (with no choice of bellower provided—only large or small bellows were used at a time), in which adult males approached the bellow broadcast, regardless of the apparent size of the caller. Our combined results suggest that adult male koalas will approach the source of a bellow regardless of the apparent size of the caller, but when given the choice, will approach the smaller one. More evidence, including multiple videos of koalas approaching and climbing onto trees with the model koala and speaker, has indicated that koalas were indeed attracted by and directly approached bellow playbacks. Similar behaviour has also been found in other animals e.g., primates [50] and birds [51].

The group bellow tests provided support to the findings from the individual bellow tests that adult male koalas preferred to approach small koala bellows. During the periods of small bellow broadcasts, adult males spent more time near speakers, compared with the time spent near the large bellow broadcast as well as the time without bellow broadcast. In both small and large bellow broadcasting periods, koalas were more active, compared with the time when bellows were not broadcast. This is further evidence for the role of koala bellows in maintaining the spatial arrangement of males in koala social systems. Although it was a relatively small sample size there were two adult males involved in the group test, that was explicit and consistent: i.e., they always chose to either approach or avoid a specific bellow broadcast without any “indifference” response. In particular, the smaller male (Dave 5.1 kg) only approached the small bellows (twice) and avoided the large bellows (twice); the larger male (Skroo 7.5 kg) approached small and large bellows nearly equally (i.e., three and four times respectively) and never showed any avoidance of them (Table 3). This may indicate a possible body weight-impact on whether an adult male chose to approach a bellow, i.e., when only one type of bellow was broadcast at a time, the small males only approach small bellows and avoid large bellows, while larger males approached both without avoidance. However, further research with a larger sample size is required to confirm these conclusions.

### 4.2. Juvenile Males

The two juvenile male koalas (males younger than three years old, *n* = 2) in this study travelled a significantly longer distance in the first move while avoiding both large and small bellows in the individual tests. These koalas never approached any bellows: in individual bellow tests, they either went in a neutral direction, avoiding both large and small bellows, or stayed at the same location. In the group tests, these juvenile males showed no approach but only avoidance of speakers when in close proximity, or stayed at the same location (Table 3). Hence, despite the small sample size, young juvenile males may tend to avoid potential rival males nearby, by either moving to avoid potential conflicts or remaining in place, relying on being undetected [52]. Although male koalas are able to produce viable sperm at two years of age [18], they only participate in breeding activities in their fourth or fifth year [18,38]. Anecdotally, one young (age 2–3) koala in our study group avoided bellows early in the study but started to approach broadcast bellows in his fourth year. Bellow avoidance behaviour could also explain the previous finding that young koalas were found using otherwise non-preferred trees while dispersing [52]. Such a conflict-avoidance mechanism based on vocalisations is common in other mammals [53,54]. Future studies with a larger sample size are needed to confirm whether bellow-avoiding behaviour of juvenile male koalas helps to explain their apparent toleration within koala social systems.

### 4.3. Females

No consistent directional movement response to bellow playbacks, hence no preference for small or large bellows, was detected for the 14 female koalas in this study. Unlike the avoidance behaviour of juvenile males, the reaction of female koalas was unpredictable across the individual and group bellow tests, because individual responses varied. Females either approached or avoided small or large bellows, or remained at the same location. Female koalas did not appear to be repelled nor attracted by male bellows in this study. Females with a dependent joey are likely to be unavailable for breeding activities, and their indifference to bellows could be expected due to the lack of specific hormones which drive behavioural responses [39]. Future research with free-ranging females that are confirmed to be in oestrus is needed to investigate whether they are affected by the broadcast of bellows from large or small koalas, but until adequate means of detecting oestrus in such individuals are established, our understanding will be based on captive behaviours.

### 4.4. First Look

The duration of the first look by koalas towards male bellow playbacks was not influenced by the size of the male caller. This is consistent with previous findings that male koalas paid equal attention to small and large koala bellows [24,30]. Although another study reported that captive oestrous female koalas would spend more time looking toward bellows indicative of a large male caller [25], we did not detect any preference in this study.

### 4.5. Implications for Koala Breeding Dynamics

The approach of adult male koalas to bellows presents solid evidence of intra-sexual competition between male koalas in the form of approaching bellows produced by other rival males. The result accords with the intra-male competitions for mating opportunities observed in captive koalas [18,20,55]. Our result also provided a reasonable explanation for the possible koala social structural plasticity [31], that the contrasting intra-male interaction frequencies observed in high-density (with frequent interactions) [31] and low-density populations (with rare interactions) [21] were due to the varied chances of male koalas being able to hear bellows from others, given that the effective distance of koala bellows was approximately 150 m [45]. This study demonstrates possible male competitions for the first time in a low-density koala population in Queensland, using bellow playbacks with free-ranging koalas. Although the importance of visual and scent cues was difficult to assess during the intra-male competition, male koalas that approached a bellow were, on occasion, recorded ascending the tree in which the model koala was placed. Given the inter-male aggressions observed in captivity [55] and in the field [31], we presume that this bellow-approaching behaviour would have led to an agonistic interaction. The scar marks on the adult male who constantly approached small bellows could also be evidence of aggressive interactions when males approach others.

Despite separately approaching broadcasts of both small and large bellows in the group bellow test, adult male koalas spent more time near small bellow speakers, and preferred to approach small bellows when they had choice (as detected in the individual test). These results support Charlton et al. [17]’s finding that koalas can perceive the body size of other male callers via the bellows produced. Similar behaviours have been reported for many other mammals [56,57,58,59]. Body size is a major factor that impacts outcomes when animals compete—larger animals primarily have a higher chance to win confrontations [53,60,61]. Therefore, it is reasonable to hypothesise that adult male koalas, detecting the bellow caller’s body size, select small males to approach due to a higher chance of winning any intra-male sexual competition.

When all the bellows broadcast are those from large males, an adult male with relatively large body size may explore and approach those bellows, even when the caller is bigger than itself (e.g., koala “Skroo” in Table 3), but an adult male of small body size may also choose to avoid the large bellows (e.g., koala “Dave” in Table 3). The avoidance behaviour of these small adult males, as well as the young males under three years old who tend to avoid all bellows, may facilitate “sneaky male” behaviour: these individuals are aware of surrounding conspecifics and avoid them, but may mate with oestrus females opportunistically. This could explain the reproductive success of smaller sires in other studies [26].

A possible outcome of bellow-approaching behaviour by adult male koalas could be to locate oestrus females. The impact of bellowing and agonistic interactions on home range size and shape has not been established in the literature, so it seems unlikely that it is a territorial activity that results in exclusive access to resources. However, male koalas have been observed bellowing before and after copulation with females [19]. Therefore, a possible explanation of the bellow-approaching behaviour of males is that adult males may use the bellows from other koalas to indicate the location of an oestrous female. Even though females cease oestrus after copulation [39], that may not hinder male’s mating attempt. Previous research suggested male koalas may not be able to tell if a female is in oestrus or not as they would attempt to mate regardless [31,62]. Another possible explanation of bellow-approaching behaviour is that males may have mistaken small male bellows for female bellows, which are sometimes observed in captivity and considered a behavioural sign of oestrus [19,39,63]. Although female bellows are distinctive from male bellows, both female and small male bellows have a higher formant frequency compared with bellows from large male koalas [17,64], and hence it may be possible for these two to be misidentified by adult males. We were unable to source any female koala bellow recordings, hence the possibility that there is mistaken bellow identity remains unresolved. In both explanations above, the target of adult males is not other male rivals but instead is females, so the approach may not necessarily end with direct conflicts between males, and hence this can also explain the low intra-male interactions detected [18,21] and why smaller males would approach males larger than themselves.

Animals often use vocalisations to claim territory [65,66,67,68], but male koalas are not typically territorial because they mainly bellow in the breeding season and their home ranges commonly overlap each other [20,69,70]. Our data do not suggest that small male koalas should rarely bellow, since they may attract the attention of larger males, so this activity may be a consequence of captivity, where isolation may reduce the cost of bellowing for such individuals. In other species, animals with a lower dominance rank make fewer calls [71,72,73,74,75]. For koalas, the relative likelihood of large or small males bellowing is unknown, but paternity analysis has indicated that body size may not determine sexual selection over time and that mate choice may be influenced by mechanisms to avoid inbreeding, whereby females select unique males rather than by body size [28,76]. We require a larger cohort of females and a better knowledge of reproductive status to determine whether bellows are a driver of female mate choice in free ranging environments. Finally, since the bellow playbacks used in this study were collected from random captive males which were unfamiliar to the testing koalas, further bellow playback studies using familiar bellows to the resident koalas are encouraged to see if free-ranging koalas respond differently to familiar and unfamiliar bellows.

## 5. Conclusions

Our data indicate that koala bellows can affect the spatial arrangement of free ranging male koalas. By choosing to approach bellows from other small males, adult males may compete with an advantage in agnostic interactions and may also benefit by locating oestrous females (since males bellow before and after copulation). Young males under three years old, and possibly adult males of a relatively small body size, may respond to bellows in a way that reduces the probability of agonistic encounters with other males larger than them, but may still facilitate opportunistic mating with oestrus females. Females, on the other hand, are suspected to be participating in the search for males to mate with when in oestrus, but our experiments could not provide direct evidence of this. With the insight into koala social strategies, our findings could facilitate koala conservation with a better understanding of the different roles adult males play before and after three years old in koala breeding and translocation programs. There is also a potential for using bellow playbacks recorded from small sized males to lure free ranging koalas into designated conservation zones, e.g., mitigation plantations and corridors connecting fragmented habitat patches.

## Figures and Tables

**Table 1:** Details of koalas from which the bellows were recorded and subsequently used in this study. Three bellow sets were used as replicates and each bellow set consisted of one small and one large bellow recording to test koala preferences.

Koala	Age (Years)	Weight (kg)	Category	Bellow Set	Source
Hudson	5	5.6	Small	1	Australia Zoo
Harley	9.5	9.3	Large	1	Australia Zoo
Dexter	5.5	5.7	Small	2	Australia Zoo
SA	Unknown	9.3	Large	2	RSPCA Wacol Hospital
Zorro	Unknown	5.7	Small	3	RSPCA Wacol Hospital
Marley	5	8.5	Large	3	Australia Zoo

**Table 2:** Group bellow test protocol showing whether the koala model was used, which bellow playback was broadcast, and the date, time and location of the broadcast in each test round. BBP D1–3 = Bellow Broadcast Positions from Day 1 to Day 3.

Test Round	Koala Model	Koala Bellow	Broadcast Hours	Date	Test Location
Control for Model	None	None	n/a	18/11/2019	BBP D1 (virtual)
				19/11/2019	BBP D2 (virtual)
				20/11/2019	BBP D3 (virtual)
Model	Yes	None	n/a	21/11/2019	BBP D1
				22/11/2019	BBP D2
				23/11/2019	BBP D3
Control for Evening bellow	None	None	n/a	30/11/2019	BBP D1 (virtual)
				1/12/2019	BBP D2 (virtual)
				2/12/2019	BBP D3 (virtual)
Evening bellow	Yes	Small	1 h (18:00–19:00)	3/12/2019	BBP D1
				4/12/2019	BBP D2
				5/12/2019	BBP D3
Evening bellow	Yes	Large	1 h (18:00–19:00)	18/12/2019	BBP D1
				19/12/2019	BBP D2
				20/12/2019	BBP D3
Control for Overnight bellow	None	None	n/a	20/03/2020	BBP D1 (virtual)
				21/03/2020	BBP D2 (virtual)
				22/03/2020	BBP D3 (virtual)
Overnight bellow	Yes	Small	6 h (18:00–00:00)	23/03/2020	BBP D1
				24/03/2020	BBP D2
				25/03/2020	BBP D3
Overnight bellow	Yes	Large	6 h (18:00–00:00)	6/04/2020	BBP D1
				7/04/2020	BBP D2
				8/04/2020	BBP D3

**Table 3:** Summary of the group bellow tests. MA = adult male, MJ = juvenile male, FS = single female, FDJ = female with a dependent joey, Acty = hourly activity level, App = approach, Avd = avoidance.

Koala	Class	Bellow	Weight (kg)	Age	Acty	Fix in 100 m	Total fix	App	Avd
Dave	MA	none	4.8	3.5	101	6	128	1	0
Dave	MA	Small	4.8	3.5	n/a	28	41	2	0
Dave	MA	Large	4.8	3.5	135	20	128	0	3
Skroo	MA	none	7.5	5.5	117	4	236	0	0
Skroo	MA	Small	7.5	5.5	147	61	120	3	0
Skroo	MA	Large	7.5	5.5	155	36	101	4	0
Tom	MJ	none	3.4	1.5	185	0	232	0	1
Tom	MJ	Small	3.4	1.5	225	0	128	0	0
Tom	MJ	Large	3.4	1.5	178	24	128	0	0
Martin	MJ	none	3.6	2	n/a	0	64	0	0
Martin	MJ	Small	3.6	2	n/a	1	42	0	1
Martin	MJ	Large	3.6	2	n/a	1	42	0	1
Jo	FS/FDJ	none	4.9	3.9	193	24	232	1	0
Jo	FS/FDJ	Small	4.9	3.9	186	43	109	1	4
Jo	FS/FDJ	Large	4.9	3.9	200	21	117	0	0
Jude	FS	none	4.1	2.9	175	0	256	0	0
Jude	FS	Small	4.1	2.9	181	1	128	1	0
Jude	FS	Large	4.1	2.9	189	18	128	1	0
Karen	FDJ	none	5.4	4.9	114	0	232	0	0
Karen	FDJ	Small	5.4	4.9	131	0	128	0	0
Karen	FDJ	Large	5.4	4.9	148	24	128	0	2
Miriam	FS	none	2	1	n/a	24	64	0	0
Miriam	FS	Small	2	1	n/a	24	64	0	0
Miriam	FS	Large	2	1	n/a	24	64	0	0

## Data Availability

The data presented in this study are available on request from the corresponding author.
